# Human Fear Acquisition Deficits in Relation to Genetic Variants of the Corticotropin Releasing Hormone Receptor 1 and the Serotonin Transporter

**DOI:** 10.1371/journal.pone.0063772

**Published:** 2013-05-22

**Authors:** Ivo Heitland, Lucianne Groenink, Elisabeth Y. Bijlsma, Ronald S. Oosting, Johanna M. P. Baas

**Affiliations:** 1 Department of Experimental Psychology and Psychopharmacology, Utrecht University, Utrecht, The Netherlands; 2 Helmholtz Research Institute, Utrecht, The Netherlands; 3 Division of Pharmacology, Utrecht Institute for Pharmaceutical Sciences, Utrecht University, Utrecht, The Netherlands; University of Wuerzburg, Germany

## Abstract

The ability to identify predictors of aversive events allows organisms to appropriately respond to these events, and failure to acquire these fear contingencies can lead to maladaptive contextual anxiety. Recently, preclinical studies demonstrated that the corticotropin-releasing factor and serotonin systems are interactively involved in adaptive fear acquisition. Here, 150 healthy medication-free human subjects completed a cue and context fear conditioning procedure in a virtual reality environment. Fear potentiation of the eyeblink startle reflex (FPS) was measured to assess both uninstructed fear acquisition and instructed fear expression. All participants were genotyped for polymorphisms located within regulatory regions of the corticotropin releasing hormone receptor 1 (CRHR1 - rs878886) and the serotonin transporter (5HTTLPR). These polymorphisms have previously been linked to panic disorder and anxious symptomology and personality, respectively. G-allele carriers of CRHR1 (rs878886) showed no acquisition of fear conditioned responses (FPS) to the threat cue in the uninstructed phase, whereas fear acquisition was present in C/C homozygotes. Moreover, carrying the risk alleles of both rs878886 (G-allele) and 5HTTLPR (short allele) was associated with increased FPS to the threat context during this phase. After explicit instructions regarding the threat contingency were given, the cue FPS and context FPS normalized in all genotype groups. The present results indicate that genetic variability in the corticotropin-releasing hormone receptor 1, especially in interaction with the 5HTTLPR, is involved in the acquisition of fear in humans. This translates prior animal findings to the human realm.

## Introduction

From an evolutionary perspective, the acquisition of fear responses enables organisms to respond appropriately to predictors of aversive events [1], [2]. In the laboratory, this process is often modeled by classical fear conditioning procedures in which an originally neutral conditioned stimulus (CS; e.g. a light) is repeatedly paired with an unconditioned aversive stimulus (UCS; e.g. an electrical shock). During the course of this acquisition process, conditioned fear responses develop towards the threat cue. As a consequence, absence of the CS may come to signal periods of safety. However, if this contingency is not acquired, threat remains unpredictable. This can result in chronic states of maladaptive anxiety in the context in which the CS is presented [3], [4]. Literature suggests that this acquisition deficit plays a crucial role in the pathogenesis of human anxiety disorders [3], [5], and large interindividual variability in fear acquisition deficits has been reported [4], [6]. As of yet, however, it remains largely unknown which neurotransmitter systems are involved in human fear acquisition deficits.

Apart from pharmacological challenges, human studies may employ the study of genetic differences as a measure of involvement of certain neurotransmitters systems, in analogy to preclinical work using knock-out mice. Both anxiety disorders [7] and fear conditioning [8] have shown strong heritability, with estimates ranging between 30% and 45%, approximately (see [9] for a review). Here, we investigate candidate polymorphisms located in regulatory regions of genes that were previously linked to fear and anxiety to assess the genetic basis of human fear acquisition deficits.

Most prominently, a polymorphism in the promoter region of the serotonin (5HT) transporter gene, often referred to as 5-HTTLPR, has been investigated with regard to fear and anxiety. The 5-HTTLPR polymorphism comprises an insertion/deletion of 43 base pairs in the 5′ promoter region of the gene, which results in either a long or a short allele. In vitro, the short allele is associated with reduced transcriptional activity [10]. On a behavioral level, short allele carriers of the 5HTTLPR (s-carriers) report heightened anxiety related personality traits [11], [12], [13], [14]. With regard to fear conditioning, psychophysiological studies have confirmed involvement of the 5HTTLPR in fear responding. An early study showed that s-carriers were more likely to show fear potentiated skin conductance responding (SCR) in a conditioning paradigm [15]. SCR is an often-used measure for fear conditioning, but reflects a rather unspecific activation of the sympathic arousal system. An established tool for more specific and cross-species assessment of fear conditioned responses is fear potentiation of the eyeblink startle reflex (FPS). It serves as an index of the basic defensive state physiology of an organism evoked by threat [16], [17], [18], rendering it an excellent tool for translational research [16], [19]. Previously, it was demonstrated that s-carriers who were able to report the CS-US contingency correctly display heightened FPS across acquisition [20]. Congruently, another study showed increased fear expression of s-carriers in an instructed fear paradigm [21].

Apart from serotonin, animal studies have implicated the corticotropin releasing factor (CRF) in fear and anxiety. CRF serves as a key neurotransmitter in physiological and behavioral responses to stress via regulation of activity in the hypothalamic–pituitary–adrenal (HPA)-axis and extra-hypothalamic regions such as the amygdala or the medial prefrontal cortex [22], [23]. Consequently, CRF has been postulated as an important factor in the pathogenesis of stress-related psychopathology such as anxiety [24]. As a potential mechanism behind this predisposition, preclinical studies posit CRF as a major factor in fear acquisition deficits. In Wistar rats, repeated local administration of CRF into the basolateral amygdala exacerbates the acquisition of cue-conditioned fear [25]. Furthermore, CRF_1_ receptor antagonists effectively block the acquisition and expression of context conditioned fear [26], [27]. In humans, relatively few large genetic studies on impact of polymorphisms in the CRF system on anxiety (pathology) have been performed as of yet, and functional effects of known polymorphisms are not yet determined. However, the concatenation of preclinical data suggesting CRF as a crucial factor in the pathogenesis of anxiety disorders has been supported in human genetic studies, in particular with regard to the corticotropin-releasing hormone receptor 1 (CRHR1) gene [28], [29]. Most notably, a single nucleotide polymorphism (rs878886) within the promoter region of CRHR1 has been linked to panic disorder [30]. In line with these results, CRHR1 antagonists have been proposed as a suitable drug for the treatment of anxiety disorders [31], [32]. Based on these findings, we aim to investigate the impact of this polymorphism mechanistically, by studying fear acquisition in healthy humans.

Apart from these direct effects of CRF on fear and anxiety, preclinical studies in rats have shown an interaction between serotonin and the corticotropin-releasing factor in the regulation of anxiety-like responses [33]. E.g., social anxiety that was induced by early social isolation can be normalized by a CRF_1_ antagonist locally infused in the dorsal raphe [33]. In addition, central CRF administration reduces activity of the serotonin neurons in the raphe and serotonin release in forebrain regions in a dose-dependent manner (see [34] for a review). Human studies investigating the CRF x serotonin interaction are much more sparse, most likely due to the lack of pharmacological agents targeting CRF that are available for the use in humans. However, recent reports have demonstrated that genetic variability in corticotropin-releasing hormone receptor 1 (CRHR1) interacts with 5HTTLPR and environmental factors on internalizing [35] and depressive symptomology [36], suggesting the interplay of both systems as an interesting target for further research.

In a related preclinical experiment in 5HTT knockout rats, we recently investigated the role of serotonin, CRF and its interplay with regard to fear acquisition deficits and its consequences. Briefly, it was shown that genetic deletion of the serotonin transporter (5HTT) was associated with blunted fear conditioned startle responses to threat cues and congruently, enhanced contextual fear responding [37]. Administration of a corticotropin-releasing factor receptor 1 antagonist during acquisition normalized the fear potentiated conditioned responding in the 5HTT knockout rats (Bijlsma et al., unpublished data). This preclinical interaction between 5HT and CRF with regard to fear acquisition may provide crucial information on the basis of interindividual variability in fear acquisition.

As a human analog to this preclinical study, we here subjected 150 healthy participants to a fear conditioning paradigm in a virtual reality environment. Eyeblink startle reflex was measured as a physiological index of fear conditioned responses, providing a direct translation of our preclinical study. To assess 5HT and CRF functioning as possible modulators of fear acquisition and fear expression, the subjects were genotyped for polymorphisms associated with fear and anxiety within regulatory regions of the serotonin transporter (5HTTLPR) and the corticotropin-releasing hormone receptor 1 (rs878886).

## Materials and Methods

### Ethics Statement

The ethical institutional review board of the University Medical Centre Utrecht approved this study, and all subjects gave written informed consent. All study procedures have been conducted according to the principles expressed in the Declaration of Helsinki.

### Participants

150 subjects (90 females, 60 males; mean age = 21.6, *SD = *2.4) were recruited via advertisements at Utrecht University. Participants filled out screening forms in which they reported to be free of any current or previous psychiatric or neurological disorder, drug or alcohol dependence, current psychoactive medication, hearing problems and color blindness. In addition, females were asked to report the number of days since the onset of their menstrual cycle. 148 out of 150 subjects were Caucasians of Western European descent, the remaining two reported to be of Asian ancestry. Participants received € 30 for their participation in the experiment. Four subjects were excluded from the final sample due to incomplete recordings of startle data (n = 1), artifacts yielding unreliable startle measurements (n = 1) and insufficient quality of isolated DNA (n = 2). The final sample therefore comprised 146 subjects between 18 and 28 years of age (87 females, 59 males; mean age = 21.7, *SD = *2.4). Data of the current sample pertaining to fear extinction are reported elsewhere [38].

### Experimental Paradigm

All subjects completed a well-established fear-potentiated startle (FPS) conditioning paradigm in a virtual reality environment adapted from [4], [6], [39] to assess the fear conditioned responding to both a threat cue and a threat context. Subjects were presented with two virtual environments. These were an apartment in a high-rise in a downtown area and a house in a suburban area (see [4] for screenshots). For each subject, one of the contexts was assigned as the threat context where shocks were administered (CXT+), whereas the other represented the safe context without shock reinforcement (CXT–). Assignment of the threat context and order of visits to the contexts was counterbalanced across subjects. An increase in background illumination (light on) with 8 seconds duration signaled when shocks could be administered in the threat context. Light-on presentations in the safe context were never followed by shock and originally implemented to assess generalization of fear. As this phenomenon was not the focus of the present study, data from this condition will be omitted for sake of brevity. Pictures from both contexts during light off and light on can be found elsewhere [39]. Subjects were presented with the virtual environments in blocks lasting 5 minutes and 25 seconds during which both contexts were visited. The beginning of each block and transitions between contexts comprised transits through a virtual metro station during which startle probes were presented to maintain startle habituation [4], [6], [38].

**Figure 1:**
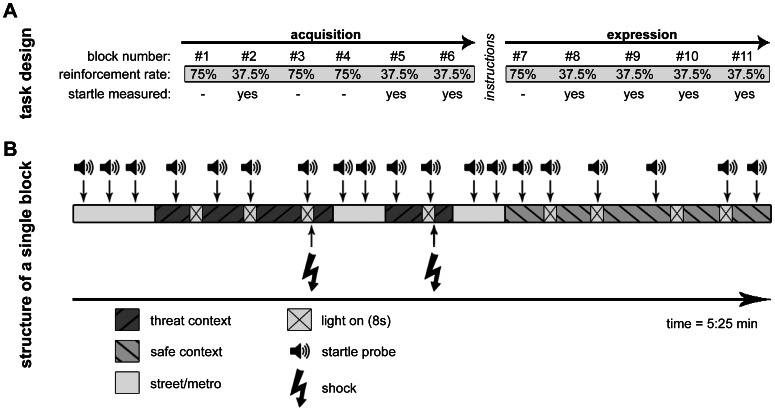
Illustration of the virtual reality fear conditioning paradigm used here. (**A**) Design of the experimental task. (**B**) Overview of the movie composition from a single acquisition block of the virtual reality fear conditioning task. Adapted from [38].

The experiment was divided into two phases (see **Figure 1** for an illustration). In the first phase, six uninstructed acquisition blocks were presented to assess the development of uninstructed conditioned responding and contingency awareness (uninstructed acquisition). During this phase, training blocks with a relatively high reinforcement rate of 75% to facilitate acquisition were alternated with testing blocks. Relatively low reinforcement rates (37.5%) during these test blocks and the transition to the next context after reinforcement prevented selective contamination of the assessment of physiological responding in the threat context due to shock sensitization [4], [6], [38]. Therefore, only startle data from test blocks (blocks 2, 5, 6 and 8–11) are reported. The uninstructed acquisition phase was followed by explicit verbal and written instructions about the contingency between threat context, threat cue and shock reinforcements to ensure contingency learning in all participants. These instructions were followed by one training block to reinforce the instructions and four testing blocks to assess instructed fear expression, the second phase of the experiment. Finally, subjects underwent an extinction phase after the fear expression phase (data are reported elsewhere; see [38]).

Throughout the experiment, startle probes were presented during three out of four light on presentations in both contexts. In addition, three startles probes were presented in absence of the light cue in each context. These are further referred to as the light off condition. As a result, each block contained three startles measurements per condition (light on/CXT+; light off/CXT+; light off/CXT–; light on/CXT–).

### Shock Administration & Workup

Electrical shocks were administered with a constant current generator (Digitimer DS7A, Digitimer Ltd., Letchworth Garden City, United Kingdom) via tin cup electrodes located approximately over the medial nerve on the inner left wrist. Before the experiment started, subjects completed a shock workup to determine individual shock intensities as described in previous publications [4], [6], [40], [41]. Intensities were adjusted per subject so that they corresponded to a level of 4 out of 5, representing ‘quite annoying/painful’.

### Startle Probe Presentation, Data Recording and Processing

Recording and amplification of the eyeblink startle reflex was performed via electromyography of the right *orbicularis oculi* muscle using a Biosemi ActiveTwo system (BioSemi Instrumentation, Amsterdam, The Netherlands). Startle probes comprised 50-ms, 105dB white noise bursts with instantaneous rise time and were delivered through headphones (Sennheiser Electronic HD202, Wennebostel, Germany). Processing of startle data was performed using Brain Vision Analyzer software (Brain Products, Gilching, Germany) according to published guidelines [42] and previous studies [4], [6], [21], [38], [41]. After segmentation of trials, artifacts were rejected and null responses identified as described previously (see [43] for procedural details and criteria). Note, that there was no association between the amount of null-responses observed during the experiment and the genetic factors under study (all *P*-values >0.28). Furthermore, it should be noted that all statistical outcomes involving startle data reported in the following (significant vs. nonsignificant) are identical with and without including the percentage of null-responses (during acquisition, during expression or during the whole experiment) as a covariate. Participants were only included in the final analysis if at least one artifact-free startle trial for each condition and each phase was recorded. One participant did not meet this criterion and was therefore excluded from further analyses. Startle data were Z-transformed per subject based on individual trial amplitudes from all startles recorded during the experiment to remove between-subjects variance in baseline startle amplitude. All statistical analyses involving startle data were conducted on Z-scores.

### Subjective Measures

Prior to the experiment, subjects filled out Spielberger’s Trait Anxiety Inventory (Dutch translation, [33]) and the neuroticism subscale of the NEO-PI-R questionnaire (Dutch translation, [44]). In addition, subjects rated their subjective fearfulness between blocks of the virtual reality fear conditioning paradigm. This was done using a visual analog scale (VAS) displayed on the computer screen together with screenshots from the pre-recorded videos representative for each condition. See [4] for examples of screenshots. The question was ‘How fearful do you feel in this situation?’ with the anchors: ‘Not at all fearful of shock’ [0] and ‘Very fearful of shock’ [100]. Two screenshots per condition were presented after each block, and an average rating was computed per condition and block. Further analysis of the data was congruent to our approach of the startle data, but data were not Z-transformed as the theoretical range of the scores was the same for every subject. In addition to these fearfulness ratings, shock contingency awareness was assessed by forced choice ratings of shock expectancy between blocks as described earlier [6].

### Genotyping

DNA was harvested by collecting buccal swaps frozen immediately at −40°C for later genotyping. Genomic DNA was extracted and purified using a QIAamp DNA Mini Kit (Qiagen, Hilden, Germany).

5-HTTLPR genotyping was performed using polymerase chain reaction ([45]; see erratum) followed by gel electrophoresis as described by [46]. This procedure visualized for each subject either two short 486 bp DNA fragments (s/s), one short and one long (529 bp) fragment (s/l) or two copies of the long fragment (l/l).

Rs878886 was genotyped using Taqman SNP Genotyping assays (ASSAY ID’s: C___7450783_10; Applied Biosystems, Foster City, CA). Subjects were classified through endpoint analysis performed on an ABI Prism 7000 (Applied Biosystems, Foster City, CA.) as either C/C homozygotes, C/G heterozygotes or G/G homozygotes. Genotyping was performed in duplicate for ∼80% of the samples without deviations.

As the taqman genotyping assay did not show any G/G homozygotes of rs878886, we decided to re-genotype the present sample in an independent lab (Department of Medical Genetics of the University Medical Center Utrecht) using Sanger sequencing to validate rs878886 genotyping. Primers for Sanger sequencing were designed using Primer3 (http://frodo.wi.mit.edu/), resulting in the forward primer sequence 5′-AGCTCATGAGTGGAAAGTCAC-3′ and the reverse primer sequence 5′-GCAAGTCTGATGATGACACC-3′. Amplification reactions were performed in a total volume of 20 µl, containing approximately 25 ng of genomic template, 50 ng of forward and reverse primer, 25 mM MgCl_2_, 2 mM dNTP’s each plus 0.8U of Taq polymerase, buffer and MQ. PCR was carried out on a thermal cycler with an initial denaturation at 94°C for 7 min, followed by 30 cycles of 30s at 94°C, 30s at 60°C, and 60s at 72°C, plus a final elongation of 4 min at 72°C. Quality and length of PCR products was checked by gel-electrophoresis (2% agarose diluted in TBE buffer) using ethidiumbromide as a staining agent and visualized under ultraviolet (UV) light. The initial PCR was successful for 140 out of 146 samples. PCR products were then purified using LSKM Multiscreen purification plates (Millipore). Sequencing of the amplicons was performed using the BigDye® Terminator v1.1 Cycle Sequencing Kit (Applied Biosystems, Foster City, CA) according to the manufacturer's specifications. Sequencing reaction products were passed through a Sephadex G-50 plate to remove unincorporated dye terminators. Sanger sequencing read-outs of rs878886 genotypes were then performed on an ABI-3730 (Applied Biosystems, Foster City, CA). All genotype calls were identical with the Taqman genotyping results we obtained. Note, that we did not detect any G/G homozygotes of rs878886 in the current sample with neither Taqman genotyping nor Sanger sequencing. For easier reading, rs878886 will further be referred to as CRHR1 (rs878886) in this report.

For genotype frequencies and statistics of the genetic polymorphisms under study, see **Table 1**. Further information on the genetic polymorphisms under study such as comparisons between previous datasets and the current dataset investigating CRHR1 (rs878886) and 5HTTLPR with regard to genotype frequencies and statistics (total *N* per dataset, minor allele frequency, Hardy-Weinberg equilibrium, *N*'s per genotype, % genotype) is presented in **Table S1**.

**Table 1:** Frequencies and statistics (Hardy-Weinberg equilibrium, linkage equilibrium and gender distribution) of the genetic polymorphisms under study are shown.

polymorphism	Hardy-Weinberg equilibrium*P* value	*N*'s			% females			*P* (gender X genotype)
CRHR1 (rs878886)	0.04	C/C	C/G	G/G	C/C	C/G	G/G	0.67
		104	42	–	59%	62%	–	
5HTTLPR	0.20	s/s	s/l	l/l	s/s	s/l	l/l	0.32
		23	79	44	61%	54%	68%	

Note: The CRHR1 (rs87886)×5HTTLPR linkage equilibrium *P* value = 0.17.

### Statistical Analyses

For clarity of presentation, planned comparisons rather than full factorial designs are reported. Of note, all statistical outcomes reported in the following (significant vs. nonsignificant) are identical when using full-factorial designs and planned comparisons. Cued fear was defined as potentiation to the threat cue within the threat context (cue FPS: light on/CXT+ vs. light off/CXT+). Contextual anxiety was defined as potentiation to the threat context in absence of the light cue (context FPS: light off/CXT+ vs. light off/CXT–).For both cue FPS and context FPS, repeated-measures ANOVA’s were conducted per phase (uninstructed acquisition and fear expression) using the contrasts stated above. CRHR1 (rs878886) genotype and 5HTTLPR genotype were included as between subjects’ factors with two levels per genotype. In addition, the CRHR1 (rs878886)×5HTTLPR interaction was entered as a between-groups factor to evaluate the a-priori hypothesis derived from preclinical research. To ascertain sufficient statistical power, we pooled the groups of s/s and s/l carriers in the group labeled ‘short allele carriers of 5HTTLPR (s-carriers)’ as commonly done regarding this polymorphism [20], [21], [38], [46].

There were no statistically significant interaction effects between the genetic factors under study and the factor block on any of the analyses performed. Details on statistics and data plots that include the factor block are therefore omitted from the main paper and can be found in **Figure S1**. Sex and age were added as covariates for all statistical comparisons that involved the genetic polymorphisms under study as commonly done in behavioral genetic research. Of note, all statistical test outcomes (significant or non-significant) reported in the following were identical with and without inclusion of these covariates.

## Results

### Descriptive Statistics

Gender distribution, mean shock intensities, baseline startle amplitudes, trait anxiety scores and neuroticism scores for all possible genotype combinations are shown in **Table 2**. Comparisons between the CRHR1 (rs878886) and 5HTTLPR genotypes with regard to trait anxiety, neuroticism, shock intensities, baseline startle amplitude and raw startle data across conditions (light on/CXT+, light off/CXT+, light off/CXT–) and phases did not reveal significant differences (all *p*-values >.10). Moreover, there were no CRHR1 (rs878886)×5HTLLPR interaction effects with regard to these measurements (all *p*-values >.10).

**Table 2:** Frequencies, N per cell, age, STAI-T, NEO-N, shock intensity, baseline startle amplitude and percentage of null-responses per experimental phase are displayed for all possible genotype combinations, and for the whole sample.

genotype		descriptives		age		STAI		NEO-N		shock intensity (mA)		baseline startle magnitude(µV)		% null-responses during acquisition		% null-responses during expression	
CRHR1 (rs878886)	5HTTLPR	*N*	% females	*M*	*SD*	*M*	*SD*	*M*	*SD*	*M*	*SD*	*M*	*SD*	*M*	*SD*	*M*	*SD*
C/C	s-carrier	76	56.6%	21.58	2.36	34.75	7.73	129.19	21.10	1.69	0.84	85.59	66.06	5.08	6.72	10.40	11.60
	l/l	28	64.3%	22.11	2.71	37.79	7.81	135.20	20.30	1.74	0.92	92.20	53.12	4.42	5.23	9.47	11.15
C/G	s-carrier	26	53.8%	21.42	2.25	35.85	7.72	133.92	25.29	1.98	1.02	84.36	43.16	4.76	6.07	7.45	8.15
	l/l	16	75.0%	21.44	2.13	39.25	8.98	134.75	20.37	1.47	0.62	78.34	54.10	5.36	6.42	4.86	5.26
whole sample		146	59.6%	21.64	2.38	36.02	7.97	131.80	21.66	1.73	0.87	85.86	58.58	4.93	6.26	9.09	10.51

### Startle Results

#### Acquisition of cue conditioning

During the uninstructed acquisition phase, significant potentiation of the eyeblink startle reflex to the threat cue was observed as indexed by contrasting startles during light on/CXT+ with startles during light off/CXT+ (cue FPS: *F*_1,142_ = 15.7, *P*<0.001, *η* ^2^ = 0.10).

**Figure 2:**
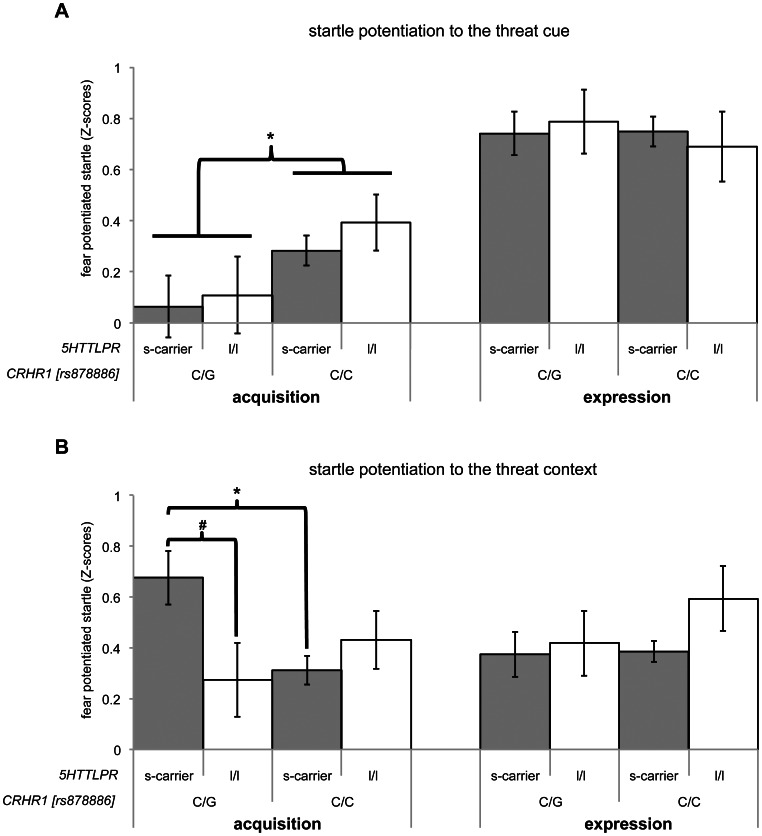
Acquisition of conditioned fear responses depends on an interaction between 5HTTLPR and CRHR1 (rs878886). Potentiated conditioned fear responses to the threat cue (**A**) and the threat context (**B**) during the acquisition phase and the expression phase are plotted as a function of both genotypes. Significant effects denoted are in (**A**) the main effect of CRHR1 on cued fear during acquisition, defined as the contrast between light on/CXT+ vs. light off/CXT+, and in (**B**) the interaction between 5HTTLPR and CRHR1 (rs878886) on contextual anxiety during acquisition, quantified as the contrast light off/CXT+ vs. light off/CXT–. Error bars display ±1 standard error of the mean *P<0.05; ^#^ *P*<0.10.

This potentiation toward the threat cue (cue FPS) was significantly modulated by CRHR1 (rs878886) genotype (*F*_1,140_ = 4.70, *P* = 0.03, *η* ^2^ = 0.03). Follow-up tests revealed that there was no fear potentiation of the startle reflex to the threat cue in the group of C/G carriers of CRHR1 (rs878886) (*F*<1), whereas C/C homozygotes showed robust fear potentiation to the threat cue (*F*_1,142_ = 37.63, *P*<0.001, *η* ^2^ = 0.27; see **Figure 2A**). The 5HTTLPR genotype and the CRHR1 (rs878886)×5HTTLPR interaction showed no significant association with cue FPS (*F*’s*<1).* For further information on the time course of these startle data across blocks, see **Figure S1**.

#### Acquisition of context conditioning

There was significant overall startle potentiation to the threat context as indexed by contrasting light off/CXT+ startles with light off/CXT– startles (context FPS: *F*_1,142_ = 67.72, *P*<0.001, *η* ^2^ = 0.32). There were no main effects of CRHR1 (rs878886; *F*<1) and 5HTTLPR (*F*_1,140_ = 2.41, *P* = 0.12) on context FPS. However, there was a significant context FPS×CRHR1 (rs878886)×5HTTLPR interaction (*F*_1,140_ = 6.77, *P* = 0.01, *η* ^2^ = 0.05). Simple main effect analysis using Tukey’s HSD Post-Hoc tests revealed that C/G carriers of CRHR1 (rs878886) who were also 5HTTLPR s-carriers showed a heightened context FPS when compared to CRHR1 (rs878886) C/C carriers with the 5HTTLPR s-carriers genotype (*p = *.02), and as a statistical trend when compared to CRHR1 (rs878886) C/G carriers with the 5HTTLPR s/s genotype (*p = *.09). See **Figure 2B** for an overview of these results and **Figure S1** for the time course of these data across blocks.

#### Expression of cue and context conditioning

Directly after the fear acquisition phase, instructions were given with regard to the shock contingency, both verbally and in writing (on-screen). Then, participants were subjected to five additional blocks to measure fear expression.

During this fear expression phase, there was significant potentiation of the startle reflex to the threat cue (*F*_1,140_ = 197.29, *P*<0.001, *η* ^2^ = 0.58) and the threat context (*F*_1,140_ = 93.6, *P*<0.001, *η* ^2^ = 0.40). However, no genetic modulations of startle activity were observed during this phase as both cue FPS and context FPS were independent of CRHR1 (rs878886) genotype, 5HTTLPR genotype and the 5HTTLPR×CRHR1 (rs878886) interaction (all *p*-values >.12; see **Figure 2**).

### Subjective Measures

#### Fear ratings

During the uninstructed acquisition phase, there was significant potentiation of subjective fearfulness to the threat cue (*F_1,142_* = 35.2, *P*<0.001, *η* ^2^ = 0.20). This potentiation was unrelated to all of the genetic factors under study (*F*’s<1.1; see **Figure S2**). Moreover, there was significant potentiation of subjective fearfulness to the threat context (*F_1,142_* = 135.3, *P*<0.001, *η* ^2^ = 0.49; see **Figure S3** for the time course of these data). This potentiation of subjective fearfulness to the threat context was unrelated to all genetic factors under study, including the 5HTTLPR×CRHR1 interaction (all *p*-values >0.10).

During the expression phase of the experiment, subjects showed significant potentiation of subjective fearfulness to the threat cue (*F_1,142_* = 186.82, *P*<0.001, *η* ^2^ = 0.57). There were no main effects of CRHR1 (*F<*1) and 5HTTLPR (*F<*1.2), but there was a significant CRHR1×5HTTLPR interaction effect (*F_1,142_* = 6.63, *P = *0.01, *η* ^2^ = 0.05; see **Figure S2**). However, post-hoc tests showed no significant differences when the four different genotype groups (CRHR1×5HTTLPR) were compared (lowest *p-*value = .06; C/G<->s-carriers vs. C/G<->l/l). In addition, there was significant potentiation to the threat context during expression (*F_1,142_* = 83.29, *P*<0.001, *η* ^2^ = 0.37). This potentiation was independent of CRHR1 genotype (*F<*1) and the CRHR1×5HTTLPR interaction (*p = *0.06), but modulated by 5HTTLPR genotype (*F_1,140_* = 4.08, *P = *0.045, *η* ^2^ = 0.03), showing an increased potentiation of subjective fear ratings to the context during the expression phase for s/s homozygotes when compared to s-carriers (see **Figure S2**).

#### Reported awareness of shock contingency

According to the criteria for the processing of forced choice data as described in [6], 128 subjects (87.7%) were aware of the ‘threat context – shock contingency’ at the end of the acquisition phase whereas 18 were unaware (12.3%). All genetic factors under study were unrelated to awareness of the ‘threat context – shock contingency’ (all P-values >0.20). With regard to the awareness of the ‘light on – shock contingency’ within the threat context, 78 subjects (53.4%) qualified as aware whereas 68 subjects (46.6%) qualified as unaware. All genetic factors under study were unrelated to awareness of the ‘light on – shock contingency’ within the threat context (all *P*-values >0.37; see **Table S2** for contingency awareness frequencies per genotype group).

## Discussion

In this human study, we investigated if genetic variability in the serotonin transporter and in the corticotropin-releasing hormone receptor 1 is associated with fear acquisition and the expression of fear. To this end, 150 healthy human subjects completed a well-established fear conditioning paradigm in a virtual reality environment and were genotyped for candidate polymorphisms in regulatory regions of the serotonin transporter (5HTTLPR) and the corticotropin-releasing hormone receptor 1 (CRHR1 - rs878886). Of note, these polymorphisms were chosen as previous reports suggested their involvement in fear and anxiety. There is converging evidence that s-carriers of 5HTTLPR are at risk for anxious personality and symptomology [11], [12], [13], [14], and presence of the G-allele of CRHR1 (rs878886) has been linked to panic disorder in a recent study that included a replication sample [30].

In the current study, the most prominent finding is the complete absence of fear conditioning to the threat cue in C/G heterozygotes of CRHR1 (rs878886), whereas solid cue conditioning was observed in C/C homozygotes. This points towards a fear-acquisition deficit in G-allele carriers of CRHR1 (rs878886). Our finding fits well with converging evidence from animal studies that suggest CRF as an important factor in fear acquisition. Importantly, a recent study showed that local infusion of CRF into the basolateral amygdala of Wistar rats enhances fear potentiation of the startle reflex to the threat cue during acquisition without affecting contextual conditioned fear suggested to model contextual anxiety [25]. It is striking to see that both in animals [25] and in this human study, there is evidence that CRF affects acquisition of uninstructed cue conditioned fear.

In accordance with these results, it was shown that increased CRF signaling within the basolateral amygdala facilitates fear acquisition [27], [47], [48]. Notably, the CRHR1 modulation in the present study was selective for uninstructed acquisition as, following the instructions, fear conditioned responding to the threat cue in the expression phase was fully reinstated in C/G carriers and was no longer distinguishable from C/C carriers. The dissociation between the presence of a genotype effect before, but not after the explicit instructions suggests that this genotype affects the acquisition of fear at the level of defensive reflexes specifically, and not so much the expression of fear after the contingencies have been disambiguated. Importantly, this CRHR1 dependent deficit of cue-driven fear acquisition was accompanied by a 5HTTLPR×CRHR1 interaction effect on fear conditioned responding to the threat context during acquisition. Carriers of the G-allele of CRHR1 (rs878886), who were also short allele carriers of 5HTTLPR, showed the highest context FPS during uninstructed acquisition. Hence, our findings indicate that these two polymorphisms (CRHR1 - rs878886 and 5HTTLPR) that both have been previously associated with fear and anxiety, are interactively involved in an exaggerated fear response to a threat context. Subjects carrying risk alleles for fear and anxiety as described in previous literature, that is both the G-allele [30] of CRHR1 (rs878886) and the short allele of 5HTTLPR [12], [13], [14], showed the highest context FPS during uninstructed acquisition. Hence, the interplay between CRHR1 and 5HTTLPR was pivotally involved in the regulation of conditioned fear acquisition to the threat context during this phase.

As argued in the introduction, failure to identify threat cues leads to unpredictability of the aversive event that follows the CS [4], [6]. Hence, a context that has been associated with threat but in which specific predictors are not responded to adaptively can induce a more chronic defensive state [49], [50]. This pattern is found in the results of 5HTTLPR s-carriers with the CRHR1 C/G genotype. Of note, this human finding is coherent with preclinical data that showed that the exacerbation of conditioned fear acquisition to the threat context in serotonin transporter knock-out rats [37] can be normalized by pharmacological treatment with a CRHR1 antagonist during acquisition (Bijlsma et al., unpublished data).

There are two studies that report an association between the short allele of the 5HTTLPR polymorphism and heightened FPS to threat cues [20], [21]. On first glance, absence of a 5HTTLPR main effect on cue FPS in the present study might therefore seem surprising. However, [21] did investigate instructed fear acquisition, which is conceptually different from uninstructed fear acquisition as measured in the current study. In [20] on the other hand, subjects unaware of the threat cue - shock contingency were excluded, which comprise a substantial part of the present sample, making the outcomes of both studies difficult to compare directly. In future studies, disentangling factors involved in fear acquisition from fear expression further would be useful to delineate whether serotonin acts differently on acquisition and expression of fear. Aside these FPS studies, several studies that used the presentation of aversive pictures to induce startle potentiation failed to find an association between 5HTTLPR genotype and startle potentiation [51], [52], [53], [54]. Moreover, recent fear conditioning studies employing functional imaging instead of the startle reflex as index of fear conditioning reported significant associations with genetic variability in 5HTTLPR [55], [56], as did a non-imaging study employing cardiovascular measurements during observational fear learning [57]. In the current study, our physiological measurement of acquisition of fear conditioning (FPS) significantly varied with genotype while our subjective measures of fear conditioning (fear ratings/contingency awareness) did not. There are several factors that might contribute to this dissociation. Cued fear acquisition in the current virtual reality fear conditioning paradigm is a relatively difficult task because no explicit instructions are given while salient contextual properties dominate, and the increase of lighting conditions that constitute the specific threat cue is relatively obscure. Explicit learning under such conditions probably depends on a variety of factors, among which higher order cognition. Other factors contributing to a reduced or divergent sensitivity of subjective measures compared to psychophysiology are intentional distortion and demand characteristics as well as individual differences in the interpretation of the subjective ratings and questions. As argued elsewhere, subjective ratings have shown to be less sensitive to pharmacological interventions [58], [59] and genetic analyses of fear conditioning [20], [21], [38], possibly because explicit knowledge about the threat contingencies and the corresponding subjective states depend more strongly on higher cognitive processes, and hence do not reflect dynamic processes in limbic structures that regulate defensive responding, such as the amygdala [9], [60].

The most important limitation of this study is the sample size. As stated earlier, there is growing concern in the field of human genetics with respect to the reliability of findings in candidate gene studies [12], [60]. However, fear conditioning, especially with fear-potentiated startle as a read-out measure, is an excellent example of a model in which all prerequisites for reliable genetic findings are satisfied [9]. Importantly, there are strong indications that individual differences in fear conditioning show strong heritability [8]. Furthermore, fear-potentiated startle measures have a relatively high ‘penetration’, meaning that the processes we measure reflect direct activation of well-described and specific neuronal circuits [9], [16]. In addition, the hypotheses can be based on strong a-priori considerations derived from preclinical work. Moreover, in this particular study the selection of the candidate genes was based on prior genetic association with anxiety (5HTTLPR: [11], [12], [13], [14]/CRHR1: [30]). And finally, with a sample size of N = 150 the study has substantial power compared to previous studies in this field. Nevertheless, replication is the true hallmark of validity, which is especially important in this exploding field. Therefore, the current study needs to be replicated with an equally large or larger sample size.

As a second limitation, our sample did not include G/G homozygotes of rs878886, and genotype distribution of this polymorphism was not in Hardy-Weinberg equilibrium (*p* = .04). While it would be interesting to analyze data that include this genotype, its occurrence is very rare [30]. The absence of G/G homozygotes in our sample may reflect self-selection of participants, as anxious individuals might be less likely to participate in experiments involving fear manipulations with shock administration. In line with this idea, the underrepresented genotype has been found to be significantly overrepresented in panic disorder patients [30]. Third, genotype groups were not matched beforehand, which lead to unequal group sizes in this study. Fourth, there is no in vivo/in vitro expression data of the CRHR1 polymorphism under study (rs878886) as of yet. This prevents directional interpretations of the present CRHR1 genotype effects on fear acquisition, and impedes the translation of preclinical CRF (ant)agonist data to the human realm. Hence, future studies on the role of CRHR1 in fear and anxiety would greatly benefit from molecular research investigating regulatory effects of rs878886 and other polymorphisms within the CRHR1 sequence. Fifth, we did not perform tri-allelic genotyping of the 5HTTLPR in the current study, meaning that genetic variability within rs25531 was not assessed. Genotyping this SNP located within the 5HTTLPR sequence allows subdivision of 5HTTLPR genotypes into A-allele or G-allele carriers [61], which potentially leads to 5HTT genotype groups that correspond more accurately to 5HTT expression levels [62]. Future studies should therefore take rs25531 into account when investigating 5HTTLPR. Sixth, we did not record oral contraceptive intake in our female subjects. This is a necessary prerequisite to investigate hormonal status effects indexed by menstrual cycle phase [63]. Therefore, we could not investigate potential effects (either as a factor or as a covariate) of female hormonal status on fear conditioning, which have been shown before [64].

Taken together, the present study suggests that the corticotropin-releasing hormone receptor 1 plays a crucial role in human fear acquisition. Moreover, the inability to appropriately condition to a danger cue that depended on CRHR1 (rs878886) was associated with heightened contextual anxiety in interaction with genetic variability in the serotonin transporter. This renders presence of the CRHR1 (rs878886) G-allele, in particular in combination with the 5HTTLPR s-allele, a risk factor with regard to inadequate responding to threat. Future studies should expand this research to further delineate the role of CRHR1, in particular in interaction with the serotonin system, in human fear acquisition.

## Supporting Information

Figure S1 **Time course of the startle response (A–D) during the virtual reality fear conditioning paradigm, as a function of condition and genotype of both 5HTTLPR and CRHR1.** In the first phase of the experiment (uninstructed acquisition; block 1–3), no instructions were given. This phase was followed by explicit instructions, and fear expression was assessed in the following phase (fear expression; block 4–7). Error bars display ±1 standard error of the mean.
(TIF)

Figure S2 **Potentiation of subjective fear ratings during acquisition and expression are shown as a function of 5HTTLPR genotype and rs878886 genotype.** Fear potentiation to the threat cue (**A**) was defined as the contrast of subjective fear ratings during light on/CXT+ vs. light off/CXT+. Fear potentiation to the threat context (**B**) was quantified as the contrast light off/CXT+ vs. light off/CXT–. Error bars display ±1 standard error of the mean.
(TIF)

Figure S3 **Time course of the subjective fear ratings (A–D) during the virtual reality fear conditioning paradigm, as a function of condition and genotype of both 5HTTLPR and CRHR1.** In the first phase of the experiment (uninstructed acquisition; block 1–3), no instructions were given. This phase was followed by explicit instructions, and fear expression was assessed in the following phase (fear expression; block 4–7). For coherence with plotting of the startle data, data points of training blocks are omitted. Error bars display ±1 standard error of the mean.
(TIF)

Table S1 **Comparisons of datasets investigating CRHR1 [rs878886] and 5HTTLPR with regard to frequencies and statistics (total** ***N*** **per dataset, minor allele frequency, Hardy-Weinberg equilibrium,** ***N*** **'s per genotype, % genotype).**
(DOC)

Table S2 **Contingency awareness frequencies at the end of acquisition are shown per genotype group.**
(DOC)
