# Supplementary figures and images for: Human Fear Acquisition Deficits in Relation to Genetic Variants of the Corticotropin Releasing Hormone Receptor 1 and the Serotonin Transporter
Source: PLoS One. 2013 May 22;8(5):e63772. doi: 10.1371/journal.pone.0063772 (PMC3661730; doi:10.1371/journal.pone.0063772)

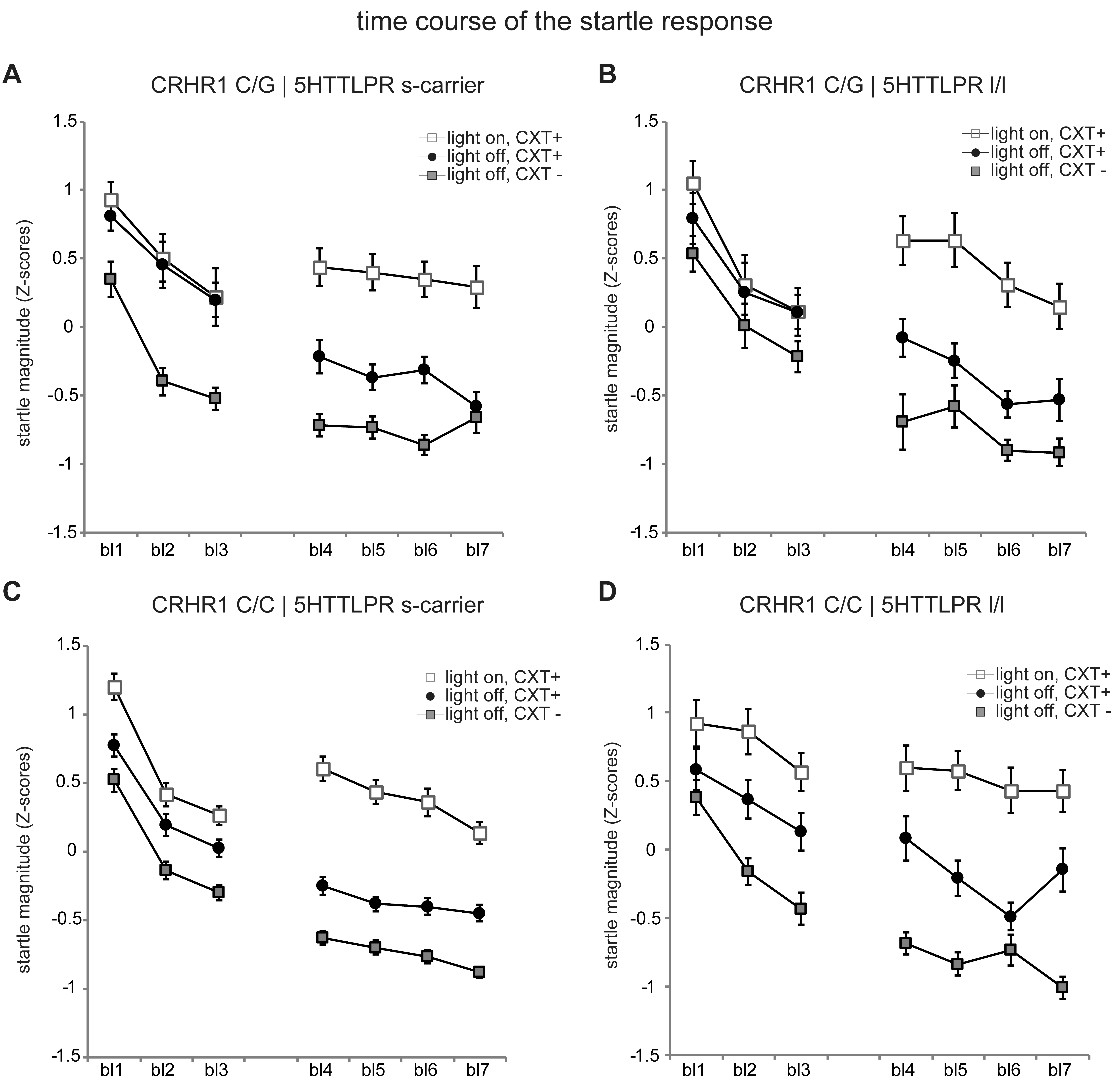

Supplement: Figure S1 — Time course of the startle response (A–D) during the virtual reality fear conditioning paradigm, as a function of condition and genotype of both 5HTTLPR and CRHR1. In the first phase of the experiment (uninstructed acquisition; block 1–3), no instructions were given. This phase was followed by explicit instructions, and fear expression was assessed in the following phase (fear expression; block 4–7). Error bars display ±1 standard error of the mean. (TIF) [file pone.0063772.s001.tif]

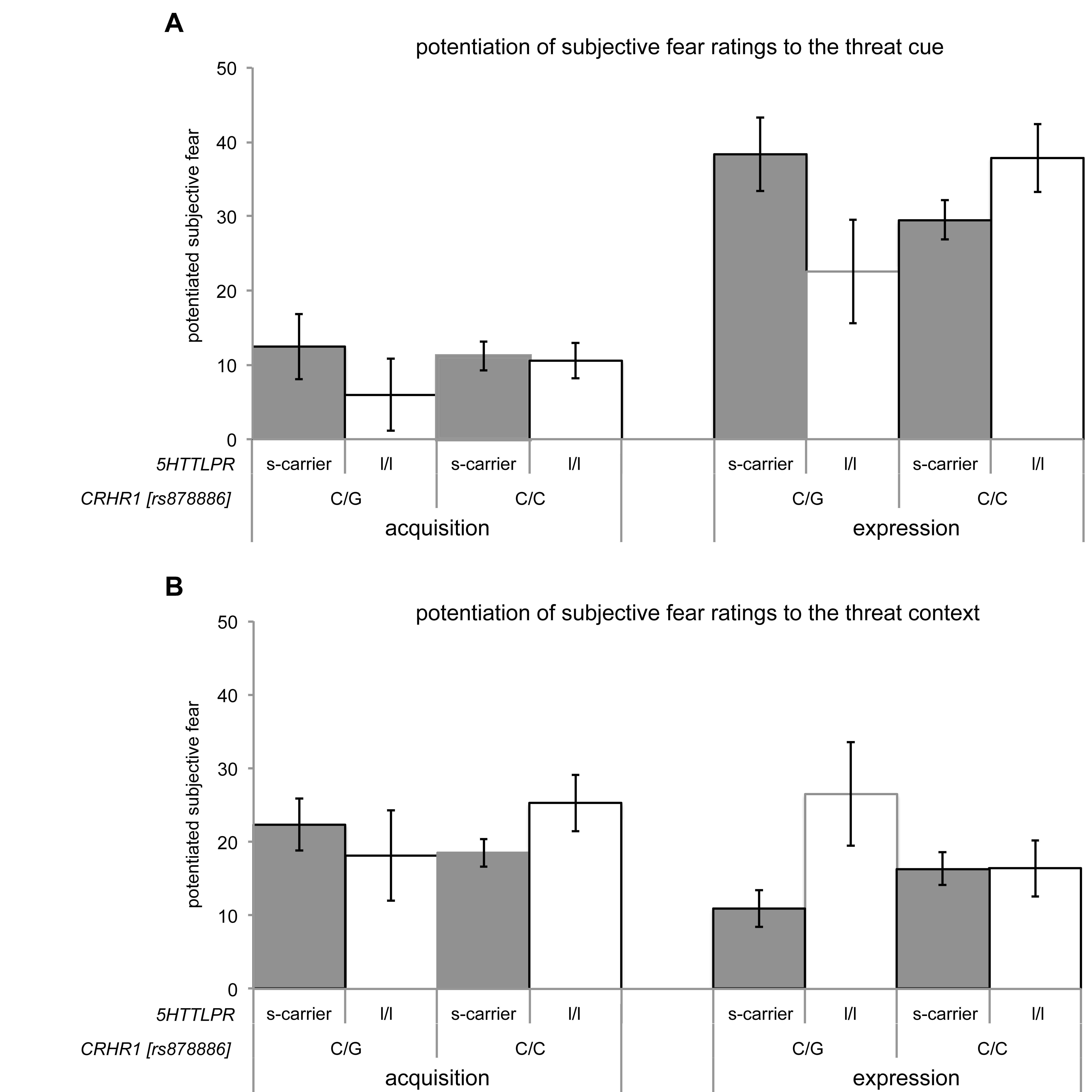

Supplement: Figure S2 — Potentiation of subjective fear ratings during acquisition and expression are shown as a function of 5HTTLPR genotype and rs878886 genotype. Fear potentiation to the threat cue (A) was defined as the contrast of subjective fear ratings during light on/CXT+ vs. light off/CXT+. Fear potentiation to the threat context (B) was quantified as the contrast light off/CXT+ vs. light off/CXT–. Error bars display ±1 standard error of the mean. (TIF) [file pone.0063772.s002.tif]

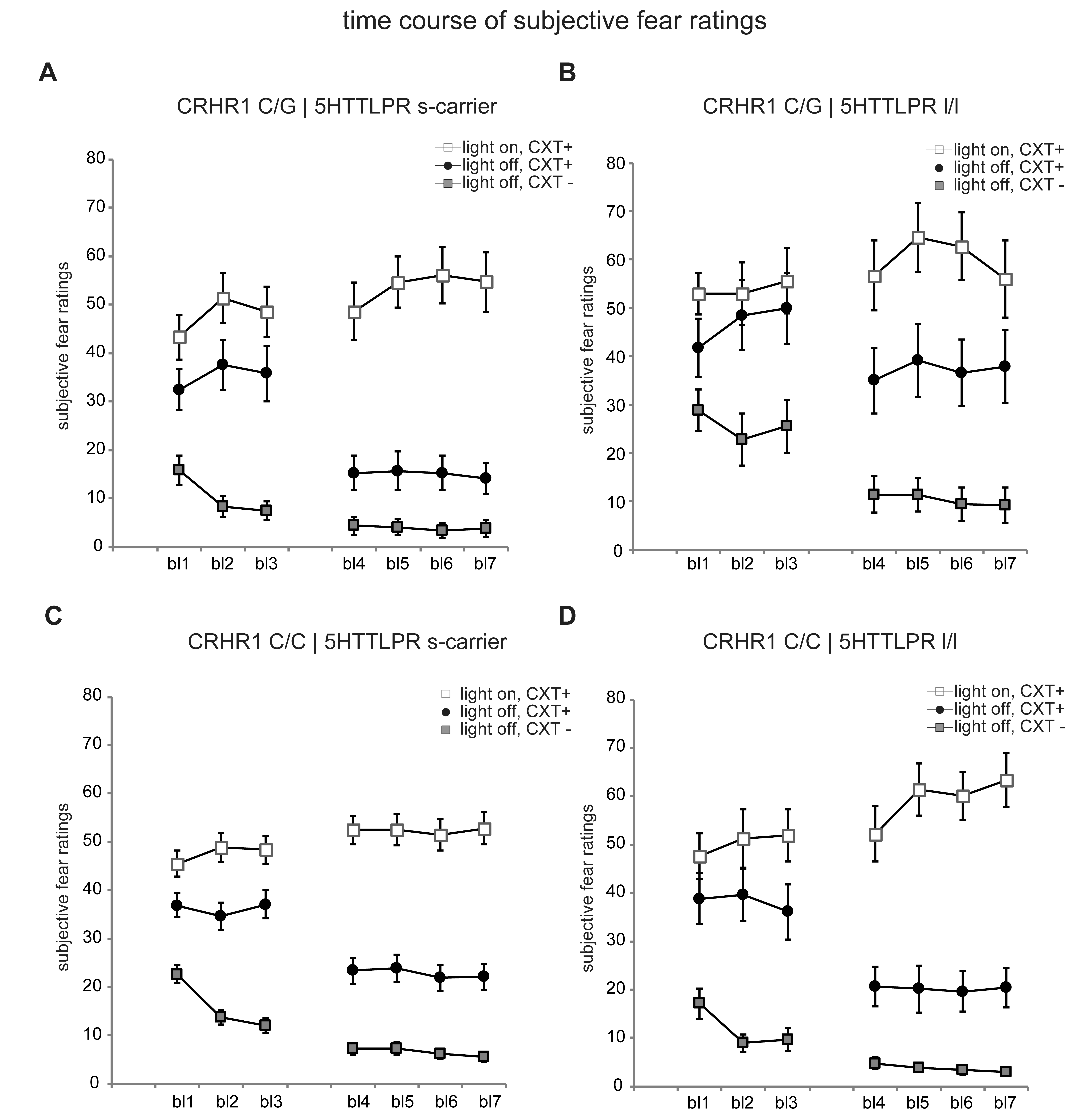

Supplement: Figure S3 — Time course of the subjective fear ratings (A–D) during the virtual reality fear conditioning paradigm, as a function of condition and genotype of both 5HTTLPR and CRHR1. In the first phase of the experiment (uninstructed acquisition; block 1–3), no instructions were given. This phase was followed by explicit instructions, and fear expression was assessed in the following phase (fear expression; block 4–7). For coherence with plotting of the startle data, data points of training blocks are omitted. Error bars display ±1 standard error of the mean. (TIF) [file pone.0063772.s003.tif]
