# Supplementary material for: Human Fear Acquisition Deficits in Relation to Genetic Variants of the Corticotropin Releasing Hormone Receptor 1 and the Serotonin Transporter
Source: PLoS One. 2013 May 22;8(5):e63772. doi: 10.1371/journal.pone.0063772 (PMC3661730; doi:10.1371/journal.pone.0063772)
Supplement: Table S1 — Comparisons of datasets investigating CRHR1 [rs878886] and 5HTTLPR with regard to frequencies and statistics (total N per dataset, minor allele frequency, Hardy-Weinberg equilibrium, N 's per genotype, % genotype). (DOC) [file pone.0063772.s004.doc]

| **Table S1.** Comparisons of datasets investigating CRHR1 [rs878886] and 5HTTLPR with regard to frequencies and statistics (total *N* per dataset, minor allele frequency, Hardy-Weinberg equilibrium, *N*'s per genotype, % genotype). | | | | | | | | | | | | |
| --- | --- | --- | --- | --- | --- | --- | --- | --- | --- | --- | --- | --- |
|  |  |  |  |  |  |  |  |  |  |  |  |  |
| Polymorphism | dataset | total *N* | Minor allele frequency | Hardy-Weinberg equilibrium *P* value | Cochran-Armitage trend test *P* value | *N*'s | | |  | % genotype | | |
| CRHR1 [rs878886] | current sample | 146 | 0.14 | 0.04 | 0.09  (*n.s.*) | C/C | C/G | G/G |  | C/C | C/G | G/G |
|  |  |  |  |  |  | 104 | 42 | 0 |  | 71% | 29% | 0% |
|  |  |  |  |  |  |  |  |  |  |  |  |  |
|  | Keck et al., 2008 (combined controls) | 749 | 0.19 | 0.23 |  | C/C | C/G | G/G |  | C/C | C/G | G/G |
|  |  |  |  |  |  | 501 | 217 | 31 |  | 67% | 29% | 4% |
|  | dbSNP | 1000 | 0.12 | - | - | C/C | C/G | G/G |  | C/C | C/G | G/G |
|  |  |  |  |  |  | - | - | - |  | - | - | - |
| 5HTTLPR | current sample | 146 | 0.43 | 0.20 | 0.56 | S/S | S/L | L/L |  | S/S | S/L | L/L |
|  |  |  |  |  |  | 23 | 79 | 44 |  | 16% | 54% | 30% |
|  |  |  |  |  |  |  |  |  |  |  |  |  |
|  | Munafo et al., 2009 | 3872 | 0.41 | 0.61 |  | S/S | S/L | L/L |  | S/S | S/L | L/L |
|  |  |  |  |  |  | 661 | 1859 | 1352 |  | 17% | 48% | 35% |
