# Supplementary material for: Human Fear Acquisition Deficits in Relation to Genetic Variants of the Corticotropin Releasing Hormone Receptor 1 and the Serotonin Transporter
Source: PLoS One. 2013 May 22;8(5):e63772. doi: 10.1371/journal.pone.0063772 (PMC3661730; doi:10.1371/journal.pone.0063772)
Supplement: Table S2 — Contingency awareness frequencies at the end of acquisition are shown per genotype group. (DOC) [file pone.0063772.s005.doc]

| **Table S2.** Contingency awareness frequencies at the end of acquisition are shown per genotype group. | | | | | |
| --- | --- | --- | --- | --- | --- |
|  | threat context - shock contingency (context) | |  | light on - shock contingency (cue) | |
| genotype group | unaware (*N*) | aware (*N*) |  | unaware (*N*) | aware (*N*) |
| C/G - S-carrier | 3 | 23 |  | 12 | 14 |
| C/C - S-carrier | 8 | 68 |  | 32 | 44 |
| C/G - L/L | 3 | 13 |  | 10 | 6 |
| C/C - L/L | 4 | 24 |  | 14 | 14 |
| total (*N*) | 18 | 128 |  | 68 | 78 |
